# Correction: Extremely low coercivity in Fe_3_O_4_ thin film grown on Mg_2_TiO_4_ (001)

**DOI:** 10.1039/c7ra90116j

**Published:** 2018-01-15

**Authors:** X. H. Liu, W. Liu, Z. D. Zhang, A. C. Komarek, C. F. Chang

**Affiliations:** Max Planck Institute for Chemical Physics of Solids Nöthnitzerstr. 40 01187 Dresden Germany xhliu@alum.imr.ac.cn; Shenyang National Laboratory for Materials Science, Institute of Metal Research, Chinese Academy of Sciences Shenyang 110016 China

## Abstract

Correction for ‘Extremely low coercivity in Fe_3_O_4_ thin film grown on Mg_2_TiO_4_ (001)’ by X. H. Liu *et al.*, *RSC Adv.*, 2017, **7**, 43648–43654.

The authors regret that two co-authors were not included in the author list for the original article. The corrected author list, to which A. C. Komarek and C. F. Chang have been added, is presented herein. The corresponding correction to the first sentence of the Acknowledgements is presented below:

“We thank L. H. Tjeng from the MPI CPfS for stimulating discussions”.

The Royal Society of Chemistry apologises for these errors and any consequent inconvenience to authors and readers.

## Supplementary Material

